# Circular RNA circVRK1 suppresses the proliferation, migration and invasion of osteosarcoma cells by regulating zinc finger protein ZNF652 expression via microRNA miR-337-3p

**DOI:** 10.1080/21655979.2021.1965695

**Published:** 2021-08-23

**Authors:** Cheng Cheng, Haoping Zhang, Zhipeng Dai, Jia Zheng

**Affiliations:** a Department of Orthopedics, Henan Provincial People’s Hospital, Zhengzhou University, Zhengzhou, China; b Department of Mini-invasive Spinal Surgery, Third Hospital of Henan Province, Zhengzhou, China

**Keywords:** CIRCVRK1, osteosarcoma, MIR-337-3p, ZnF652

## Abstract

Circular RNA is an innovative kind of endogenous non-coding RNA, which could take part in tumorigenesis. Nonetheless, the potential molecular mechanisms of circVRK1 in the progression of osteosarcoma remain unresolved. In the current study, we initially investigated circVRK1 levels in osteosarcoma clinical samples and cell lines by qRT-PCR analysis and northern blot assay. RNase R treatments, RNA stability assay and nucleoplasmic separation assay were conducted to identify the characteristics of circVRK1. We adopted CCK-8, colony formation, wound-healing, and transwell assays to assess the biological effects of circVRK1 on the proliferation, migration, and invasiveness of osteosarcoma cells in vitro. We then constructed a xenograft model in nude mice to confirm the suppressive role of circVRK1 in vivo. Moreover, dual-luciferase reporter, RNA immunoprecipitation, and RNA pull-down assays were utilized to elucidate the underlying molecular mechanisms mediated by circVRK1. We demonstrated that circVRK1 was a stable circular transcript localized in the cytoplasm of osteosarcoma cells, and the down-regulation of circVRK1 in osteosarcoma tissues was related to poor outcome of patients. Meanwhile, over-expressed circVRK1 obviously restrained the growth, migration, and invasion of osteosarcoma in vitro and in vivo. Mechanistically, circVRK1 was assumed to be a microRNA sponge for miR-337-3p, and ZNF652 was the downstream gene of miR-337-3p. CircVRK1 overexpression or miR-337-3p knockdown accelerated ZNF652 expression, and up-regulated miR-337-3p efficiently abolished the promotion of ZNF652 induced by circVRK1. Moreover, rescue experiments have proved that circVRK1 inhibits the progression of osteosarcoma by modulating the miR-337-3p/ZNF652 axis. Therefore, we conclude that circVRK1 promotes ZNF652 expression by sponging miR-337-3p. CircVRK1 serves as a molecule sponge for miR-337-3p and mediates the ceRNA network to promote the expression of ZNF652, thus suppresses osteosarcoma proliferation, migration and invasion.

## Introduction

One of the malignant bone tumors, osteosarcoma, was commonly seen in children and adolescents, and it has the characteristics of high morbidity and mortality [1–3]. Marina et al. reported approximately 400 new cases of osteosarcoma per year in the United States [4]. At present, with the advance of surgical technology and the application of neoadjuvant chemotherapy drugs [2,5], the overall event-free survival rate could reach 60–70% [6,7]. However, osteosarcoma is prone to distant metastasis, mainly to the lung [8–10]. Once osteosarcoma patients have distant metastasis, the five-year survival rate has decreased to lower than 40% [7,11]. Therefore, exploring the potential mechanism of osteosarcoma development is urgently required for ameliorating the prognosis of osteosarcoma patients.

As a kind of non-coding RNA, circRNA is usually originated from the exon of genes and is formed by ‘direct back splicing’ or ‘exon skipping’ [12]. Although circRNA has been found for approximately forty years, its functions in physiological and pathological processes remain largely unknown. In the recent years, with the advancement of high-throughput sequencing technologies, thousands of circRNAs have been identified in the human diseases. Most of them are featured by evolutionary conservation, high stability, and tissue-specific expression [13–15]. In human cancers, substantial researches have confirmed that circRNAs play a critical role in tumor growth, metastasis, and chemoresistance. Yan et al. revealed that circPVT1 accelerates osteosarcoma metastasis by the miR-526b/FOXC2 axis [16]. Chen et al. reported that the upregulation of hsa_circ_0008792 inhibited osteosarcoma progression by modifying miR-711/ZFP1 signals [17]. Furthermore, Zhang et al. demonstrated that has_circ_0102049 suppressed the progression of osteosarcoma via modulating miR-520 g-3p/PLK2/TAp73 axis [18]. These studies suggested that aberrant circRNAs expression might be encouraging diagnosis biomarkers and therapeutic target for osteosarcoma. CircVRK1 originates from chromosome 14 and regarded as a tumor inhibitor in the various cancers [19,20]. However, its role in the malignant characterization of osteosarcoma is undiscovered.

The theory of competing endogenous RNA (ceRNA) is essential for circRNA to manifest its biological functions in eukaryotic cells. CircRNA competitively binds to microRNA (miRNA) through the microRNA response element (MRE), releasing messenger RNA (mRNA) which is bound to miRNA, thereby regulating the expression of mRNA in cells [12,21,22]. For example, Liu et al. indicated that hsa_circ_0000228 is an oncogenic circRNA, which participates in promoting cervical cancer progression via sponging miR-195-5p to modulated LOXL2 expression [23]. Zeng et al. found that miR-145 was identified as a downstream target mediating the effect of circBCL11B by targeting LASP1 in oral squamous cell carcinoma [24]. Besides, growing evidence indicated that miR-337-3p affected the proliferation, migration, and invasion abilities of cancer cells, such as osteosarcoma, lung cancer and epithelial ovarian cancer [25–27]. Importantly, many studies have confirmed that some circRNAs could serve as a miR-337-3p sponge to assume a regulatory function [28,29]. Furthermore, a novel zinc-finger protein 652 (ZNF652) has been described as a transcriptional repressor and directly repressed tumor promoter in breast cancer [30,31]. However, whether miR-337-3p and ZNF652 are affected by the expression of circVRK1 in osteosarcoma is unidentified.

In the current study, we hypothesized that circVRK1 assumes an important role in the progression of osteosarcoma. The research goal is to reveal the biological functions and potential molecular mechanism of circVRK1 regulating the progression of osteosarcoma. We initially tested circVRK1 levels in the osteosarcoma tissues and cells. Meanwhile, we analyzed the relationship between circVRK1 and patients’ clinicopathological characteristics. Furthermore, we assessed the functions of circVRK1 on the cell growth, migration and invasiveness of osteosarcoma. Finally, we revealed the underlying mechanism that circVRK1 sponged miR-337-3p and accelerated the ZNF652 expression.

## Materials and methods

### Clinical samples

From January 2013 to June 2017, 57 osteosarcoma cases who accepted surgical treatment in the department of orthopedics of Henan Provincial People’s Hospital were selected. Collecting osteosarcoma tissues and matched adjacent noncancerous tissues. Clinical samples were placed in RNAlater™ Stabilization Solution (ThermoFisher, CA, USA) and frozen in liquid nitrogen within 10 minutes after excision from the body. All patients were followed up until March 2021, with no lost cases. The present study was approved by the Clinical Ethics Committee of Henan Provincial People’s Hospital, and informed consent was obtained from patients before surgical operation.

### Cell culture

Human osteosarcoma cell lines U-2OS, MG-63, MNNG/HOS, Saos-2, 143B, and normal osteoblast cell-line hFOB1.19 were obtained from Zhong Qiao Xin Zhou Biotechnology Co., Ltd. (Shanghai, China). The method of cell culture referred to the previous research [32]. The osteosarcoma cells and osteoblasts were cultured in complete DMEM/F12 medium (Gibco, CA, USA) containing 10% fetal bovine serum (Gibco, CA, USA), and the culture conditions for osteosarcoma cells was 37°C, 5% CO_2_ and 99% relative humidity, while the culture temperature for hFOB1.19 was 33.5°C.

### Cell transfection

In order to achieve circVRK1 overexpression in osteosarcoma cells, vectors containing full-length of circVRK1 and corresponding negative control empty vectors were constructed by GenePharma (Shanghai, China). Small interfering RNA (siRNA) targeting ZNF652 (si-ZNF652, 5'-GUAGAGAAAGUCAGCGUUA-3'), siRNA negative control (si-NC, 5'-CUUGCCGCGCGUGUCUUGU-3'), miR-337-3p mimics (miR-337-3p, 5'-CUCCUAUAUGAUGCCUUUCUUC-3'), mimics negative control (miR-NC, 5'-UUGUACUACACAAAAGUACUG-3'), miR-337-3p inhibitors (anti-miR-337-3p, 5'-GAAGAAAGGCAUCAUAUAGGAG-3'), and inhibitors negative control (anti-NC, 5'-UUCUCCGAACGUGUCACGUTT-3') were obtained from RiboBio Co., Ltd. (Guangzhou, China). Lipofectamine 3000 reagent (Invitrogen, CA, USA) was employed to conduct cell transfection according to the manufacturer ‘s guidelines and previous article [33].

### Western blot

The experimental method of western blot referred to the previous literature [34]. We adopted RIPA lysate containing cocktail inhibitors (Beyotime, Shanghai, China) to extract total protein. The BCA kit (Beyotime, Shanghai, China) was used to determine the protein concentration. The total lysates of each simple were separated by 4–20% gradient sodium dodecyl sulfate (SDS)-polyacrylamide gel (Bio-Rad, CA, USA), followed by transblotting onto the nitrocellulose membranes. After using the nonfat milk to perform the nonspecific blinding, we incubated the membranes overnight with the following primary antibodies: anti-ZNF652 (ab126880, Abcam, Cambridge, UK), anti-GAPDH (ab8245, Abcam). Following three times washing by TBS-T solution, appropriate horseradish peroxidase-conjugated secondary antibody was used to develop blots at room temperature for 1 hour. Finally, protein levels were analyzed by the ECL analysis system (Bio-Rad, CA, USA).

### Quantitative real-time PCR (qRT-PCR)

Total RNA in tissues and cells was extracted by TRIzol reagent (Beyotime, Shanghai, China) and quantified by NanoDrop 2000 (ThermoFisher, CA, USA). Nuclear and cytoplasmic RNAs were separated using the PARIS^TM^ Kit (Invitrogen, CA, USA) according to the standard instruction. The primers were constructed from Sangon Biotech (Shanghai, China) and the sequence of primers is shown in Table 1. cDNA was synthesized by using the PrimeScript RT Reagent Kit (Takara, Shiga, Japan), and MicroRNA Reverse Transcription Kit (Takara, Shiga, Japan) was used to perform miRNA reverse transcription. qRT-PCR was carried out by the SYBR Primer-Script RT-PCR kit (Takara, Shiga, Japan) with the QuantStudio™ 6 Flex PCR system (ThermoFisher, CA, USA). The relative expression levels of each gene were analyzed by the 2^−ΔΔCt^ method. Additionally, the PCR products of circVRK1 were performed Sanger sequencing by Sangon Biotech (Shanghai, China) to validate the back-splice junction sequence of circVRK1. Detailed, PCR products were resolved and size separated on 1.5% agarose gel supplemented with YeaRed Nucleic Acid Gel stain (Yeasen, Shanghai, China) and confirmed by Sanger sequencing with standard methods [19].

**Table 1.** The primers used in this study are listed as follows

	Sequence (5'-3')
Primers	
CircVRK1 Forward	GAACCTGGTGTTGAAGATACGG
CircVRK1 Reverse	AATCCTACTTTCCATTCCTTTTTTG
VRK1 Forward	AATTGGGGCAACACGAAAGC
VRK1 Reverse	CATACACTCCGGGATCTGGC
STAT3 Forward	AAACTGCTTGCCTTGACCAC
STAT3 Reverse	CGCCTTGCCTTCCTAAATAC
RUNX1T1 Forward	CCATTGCCCACCACTA
RUNX1T1 Reverse	CCACTCTTCTGCCCATT
ZNF652 Forward	CTTCACCAGCAAACAGACTGTGAA
ZNF652 Reverse	TTCTTTTCTGCATATCCATGGACG
ZDHHC23 Forward	GTCGGGCAGTCTCAACAATC
ZDHHC23 Reverse	TCCTCACACAGATGCCACAT
PCGF2 Forward	GCATCTTGCCAAGTTTCTCC
PCGF2 Reverse	TCTGCAGGCAGTTCAAGCTA
APOL6 Forward	GATGTGCGAACTGGACACAG
APOL6 Reverse	CATAGGGGGCGTCAAACAG
GAPDH Forward	CAAGGCTGAGAACGGGAAG
GAPDH Reverse	TGAAGACGCCAGTGGACTC
miR-337-3p Forward	CGCGCTCCTATATGATGCCT
miR-337-3p Reverse	GTGCAGGGTCCGAGGT
U6 Forward	CGCTTCGGCAGCACATATAC
U6 Reverse	TTCACGAATTTGCGTGTCAT

### Northern blotting

Northern blotting was referred to the method used in the previous research [35]. Briefly, RNAs were isolated from osteosarcoma and normal samples using TRIzol reagent. CircVRK1 and 18S probes for northern blotting were achieved using the Biotin RNA labeling mix (Roche, Basel, Switzerland). The RNA samples were separated by electrophoresis and were transferred to NC membranes, which were then incubated with the hydration buffer containing the probes. Finally, the RNA signal was detected using the Chemiluminescent Nucleic Acid Detection Module (Thermo Scientific, CA, USA).

### RNA stability assay

To compare the stability of circular isoform and linear isoform of VRK1, osteosarcoma cells were exposed to 5 μg/ml actinomycin D (Biosharp, Beijing, China) for 8, 16 and 24 hours to block transcription. For RNase R digestion, 2 μg of total RNA was incubated with 8 U RNase R (Sigma-Aldrich, MO, USA) for 30 minutes at 37°C. The levels of transcripts were measured by qRT-PCR analysis [32].

### Cell proliferation activity assay

We used Cell Counting Kit-8 (CCK-8, Dojindo, Kumamoto, Japan) to detect cell growth ability and the method referred to the previous study [36]. Briefly, osteosarcoma cells were seeded in 96-well plates at 2000 cells per well and cultured for 24,48 and 72 hours. After the prescribed time, we replaced the complete medium with 100 μl serum-free medium and 10 μl CCK-8 solution, then incubated at 37°C for 2 hours in the dark. A microplate reader (ThermoFisher, MA, USA) was used to measure and evaluate the cell proliferation viability at an absorbance of 450 nm.

### Colony formation assay

The transfected cells were added to 12-well plates at 100 cells per well and cultured under standard conditions. Fourteen days later, the colonies on the plates were soaked in 4% paraformaldehyde for 15 minutes and dyed with crystal violet solution (Beyotime, Shanghai, China) for 5 minutes, taking a general view of colonies with an SLR camera (Nikon, Tokyo, Japan).

### Transwell assay

The cell invasiveness was examined with the 8-μm pores transwell chamber (BD Falcon, NJ, USA) which was pre-coated with Matrigel (BD Falcon, NJ, USA). According to previous study [37], 3 × 10^4^ cells were cultured in the top chamber with 300 μl serum-free medium, and in the bottom chamber with 500 μl complete DMEM/F12 medium. After incubating for 48 hours, we removed unpassed cells on the top of the membrane with a cotton swab, then washed chambers with PBS followed by fixing with polyformaldehyde, lastly staining with crystal violet solution. After staining, the membrane was cut from the chamber and placed on the slide. An inverted phase microscope (Olympus, Tokyo, Japan) was adopted to observe and count the cells, which were transferred through the polycarbonate membrane.

### Wound-healing assay

We carried out the wound-healing assay with reference to the previous research methods [37]. U-2OS and MNNG/HOS cells were cultured in the 6-well plates. A horizontal line with the same width was drawn at the bottom of the plate with a 100 μl pipette when the fusion degree reached 90%. After 24 hours of culture with serum-free medium, the scratch width was observed and photographed by an inverted microscope.

### Dual-luciferase reporter assay

The fragment of circVRK1 and the 3'-UTR of ZNF652 mRNA containing miR-337-3p binding sites were cloned into the PGL3 luciferase reporter vector (GenePharma, Shanghai, China) to construct the wild-type (WT) luciferase reporter vectors circVRK1-WT and ZNF652 3'-UTR-WT, respectively. The mutant (Mut) vectors circVRK1-Mut and ZNF652 3'-UTR-Mut were generated by mutating the binding sites of miR-337-3p in the circVRK1 sequence and the 3'-UTR of ZNF652 mRNA. According to the method of previous study [38], osteosarcoma cells were planted in 48-well plates and co-transfected with the above vectors and miR-337-3p mimics. After 48 hours, the activity of luciferase was detected by the dual-luciferase assay system (Promega, WI, USA).

### RNA pull-down assay

RNA pull-down assay was conducted using the technique, which Zhang et al. reported [39]. U-2OS and MNNG/HOS cells were incubated with 3' terminal-biotinylated-circVRK1 probe or oligo probe (RiboBio, Guangzhou, China) for 48 hours. Later, we collected cells and lysed in lysis buffer. After washing three times with pre-cold lysis buffer, the cell lysates were incubated with M-280 streptavidin magnetic beads (Sigma, MO, USA) for 3 hours at 4°C. In order to assess the relative enrichment levels of miR-337-3p, the bound RNAs were conducted by qRT-PCR assay.

### RNA immunoprecipitation assay (RIP)

To further confirm the interaction between circVRK1 and miR-337-3p, the RIP assay was performed with the Magna RIP RNA Binding Protein Immunoprecipitation Kit (Millipore, MA, USA). Briefly, 1 × 10^7^ cells were lysed with RIP lysis buffer, then cell lysates, A/G immunomagnetic beads (Millipore, MA, USA), anti-AGO2 (ab186733, Abcam) or anti-IgG antibody (ab172730, Abcam) were premixed in immunoprecipitation buffer at 4°C overnight to immunoprecipitate AGO2-RNA and IgG-RNA complexes. Thereafter, complexes were digested by protease K (Sigma, MO, USA) and remained RNA steps into routine qRT-PCR for measuring circVRK1 and miR-337-3p levels [40].

### Xenograft model

We purchased six 4-week-old female BALB/c nude mice from Charles River Laboratory (Beijing, China) and randomly clustered into two groups and subcutaneously injected with stable circVRK1 overexpression or control MNNG/HOS cells. The width (W) and the length (L) of tumors were estimated using a caliper every week. We use the formula V = (W^2^ × L)/2 to calculate the size of the tumor. After 28 days, we sacrificed all the mice through cervical dislocation and resected the xenograft tumors, weighed and photographed. Moreover, tumor tissues were harvested for hematoxylin–eosin (HE) staining as well as qRT-PCR analysis. All animal experiments were carried out as per the guidelines of the Ethics Committee of Henan Provincial People’s Hospital.

### Statistical analysis

All data were analyzed and depicted using GraphPad Prism 7.0 software (GraphPad Inc., CA, USA). The difference between the two groups was compared with the student’s *t-*test. Among the multiplex groups, one-way analysis of variance (ANOVA) was adopted. The Kaplan-Meier curve and log-rank test were used to analyze the effect of circVRK1 level on the overall survival status of osteosarcoma patients. The association between circVRK1 expression level and clinical features was analyzed via χ2 analysis. The linear association between circVRK1 and miR-337-3p, ZNF652 and circVRK1, ZNF652 and miR-337-3p were tested by Pearson’s Correlation Coefficient. The values with *P* < 0.05 was considered to be statistically significant.

## Results

This study aimed to reveal the expression, biological function and potential molecular mechanism of circVRK1 in osteosarcoma. We hypothesized that circVRK1 assumes an essential role in the progression of osteosarcoma. Based on our results of in vitro and in vivo experiments, we demonstrated that circVRK1 was remarkably down-regulated in osteosarcoma tissues and cell lines, and circVRK1 could adsorb miR-337-3p to up-regulate ZNF652 expression to suppress osteosarcoma progression.

***CircVRK1 is down-regulated in osteosarcoma tissues, cells and correlated with the prognosis of osteosarcoma patients***

**Figure 1.**
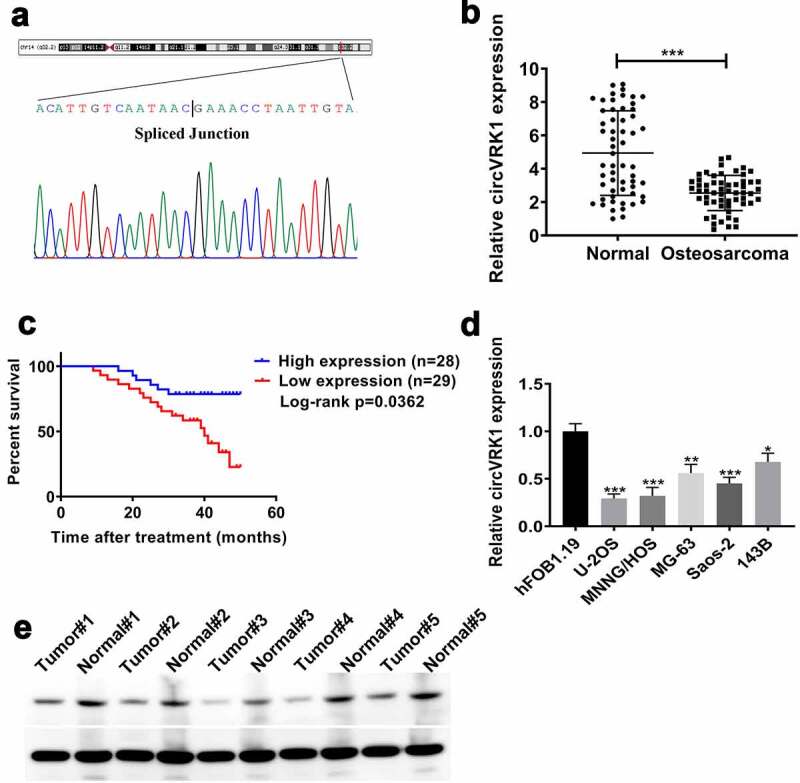
CircVRK1 was frequently repressed in osteosarcoma and correlated with the prognosis of patients with osteosarcoma

Initially, the head-to-tail junction sequences of circVRK1 were identified by Sanger sequencing (Figure 1a). The expression of circVRK1 was determined by qRT-PCR in 57 pairs of osteosarcoma tissues and matched adjacent tissues. The results indicated that circVRK1 levels were obviously more down-regulated in osteosarcoma tissues than in noncancerous tissues (Figure 1b). Meanwhile, according to the median of circVRK1 level, we divided 57 cases into two groups, namely, CircVRK1 High group (n = 28) and CircVRK1 Low group (n = 29). The Kaplan-Meier curve indicated that patients with low level of circVRK1 had a worse prognosis (Figure 1c). Besides, low expression of circVRK1 was associated with distant metastasis, while there was no significant correlation between circVRK1 expression and patients’ gender, age, tumor location, pathological grade, chemosensitivity, serum alkaline phosphatase and radiological feature (Table 2). Furthermore, we used qRT-PCR to analyze the levels of circVRK1 in osteosarcoma cells and osteoblast cells. Consistent with clinical samples, osteosarcoma cell lines displayed decreased abundance of circVRK1 than hFOB1.19 cells (Figure 1d). However, the expression levels of circVRK1 in different osteosarcoma cell lines were different, the levels of circVRK1 in MG-63 and 143B cells were higher than those in U-2OS, MNNG/HOS and Saos-2 cells. The reason may be related to the different degree of differentiation and tissue resource of different osteosarcoma cell lines. Therefore, we selected MNNG/HOS and U-2OS cells for further experiments considering they had relatively lower circVRK1 expression. We then measured circVRK1 expression through Northern blotting in five pairs of osteosarcoma tissues and normal tissues. The results indicated that circVRK1 levels were lower in tumor tissues (Figure 1e). Collectively, these data suggested that circVRK1 is down-regulated in osteosarcoma and might be assumed as an anti-tumor gene in osteosarcoma.

**Table 2.** Characteristics of patients with osteosarcoma

Variables	CircVRK1 High (n = 28)	CircVRK1 Low (n = 29)	*P* value
Gender			0.494
Male	17 (60.71%)	15 (51.72%)	
Female	11 (39.29%)	14 (48.28%)	
Age (years)			> 0.99
Median	17.2	16.0	
Range	9–22	7–23	
Anatomical site			0.786
Femur	12 (42.86%)	10 (34.48%)	
Tibia	9 (32.14%)	13 (44.83%)	
Humerus	5 (17.86%)	3 (10.34%)	
Pelvis	1 (3.57%)	1 (3.45%)	
Other	1 (3.57%)	2 (6.90%)	
Histologic subtype			0.772
Osteoblastic	16 (57.14%)	13 (44.83%)	
Chondroblastic	4 (14.29%)	6 (20.69%)	
Fibroblastic	1 (3.57%)	2 (6.90%)	
Telangiectatic	5 (17.86%)	4 (13.79%)	
Other	2 (7.14%)	4 (13.79%)	
Histologic grade			0.705
III	16 (57.14%)	18 (64.29%)	
IV	12 (42.86%)	11 (39.29%)	
Metastasis			0.012
With	11 (39.29%)	21 (72.41%)	
Without	17 (60.71%)	8 (27.59%)	
Chemosensitivity			0.308
Sensitive	20 (71.43%)	24 (82.76%)	
Insensitive	8 (28.57%)	5 (17.24%)	
Serum ALP level	875.39 ± 22.63 U/L	826.73 ± 27.28 U/L	0.661
Radiological feature			0.412
Central osteosarcoma	15	20	
Parosteal osteosarcoma	6	3	
Periosteal osteosarcoma	7	6

(A) The sequence of spliced junction of circVRK1 was validated by Sanger sequencing. (B) qRT-PCR for determining the level of circVRK1 in osteosarcoma tissues and matched adjacent tissues (n = 57). (C) Kaplan-Meier analyzed the relationship between circVRK1 level and osteosarcoma patients’ overall survival. (D) The expression of circVRK1 in osteosarcoma cell lines and osteoblast line was detected by qRT-PCR. (E) Northern blotting analysis of circVRK1 expression in osteosarcoma tissues and paired normal tissues. *P < 0.05, **P < 0.01, ***P < 0.001.

### CircVRK1 is localized in the cytoplasm of osteosarcoma cells

**Figure 2.**
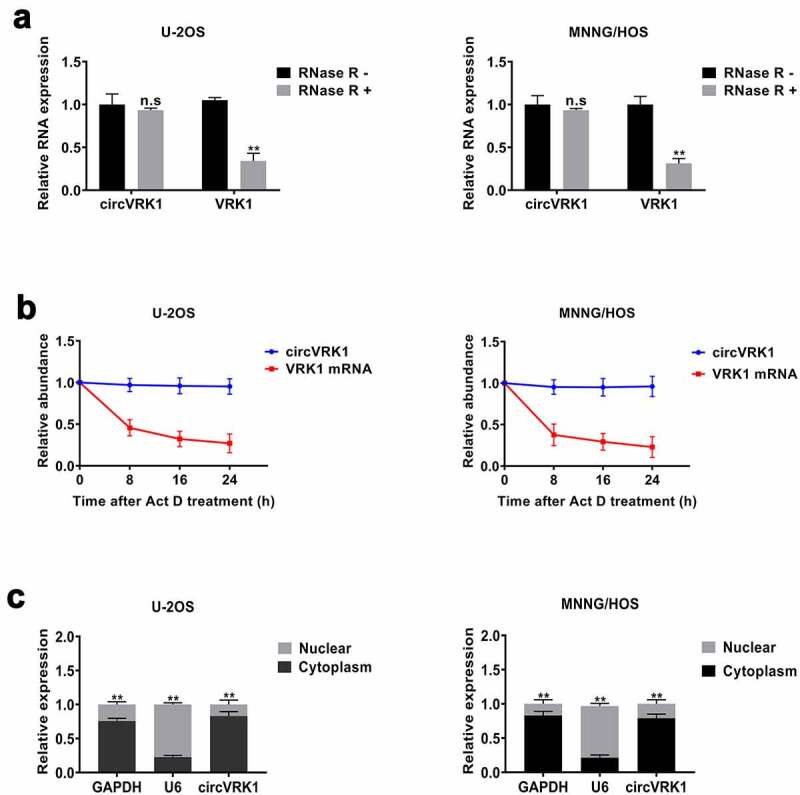
Characteristics of circVRK1

To identify the features of circVRK1, we conducted a series of experiments. First, we treated osteosarcoma cells with RNase R and the transcripts level of circVRK1 and VRK1 mRNA were tested with qRT-PCR. The data showed that the circular form of VRK1 could be preserved after RNase R treatment. In contrast, the linear form was digested by RNase R, which verified that the circular structure of circVRK1 could resist to RNase R (Figure 2a). Moreover, RNA stability assay was performed to compare the stability of circular isoform and linear isoform of VRK1 in osteosarcoma cells, and the results illustrated that circVRK1 was more stable than VRK1 mRNA (Figure 2b). Besides, the results of the nucleoplasmic separation assay determined that circVRK1 was preferentially localized in the cytoplasm (Figure 2c). In summary, these results demonstrated that circVRK1 has a stable circular structure and is located in the cytoplasm of osteosarcoma cells.

(A) qRT-PCR evaluated the relative abundance of circVRK1 and VRK1 mRNA in U-2OS and MNNG/HOS cells after being treated with RNase R. (B) The abundance of circVRK1 and VRK1 mRNA in U-2OS and MNNG/HOS cells treated with Actinomycin D was detected by qRT-PCR at indicated time point. (C) qRT-PCR was utilized to assess the contents of circVRK1, U6 and GAPDH in the cytoplasm or nucleus of osteosarcoma cells. **P < 0.01, n.s is no significance.

### CircVRK1 suppresses the proliferation, migration and invasion of osteosarcoma cells

**Figure 3.**
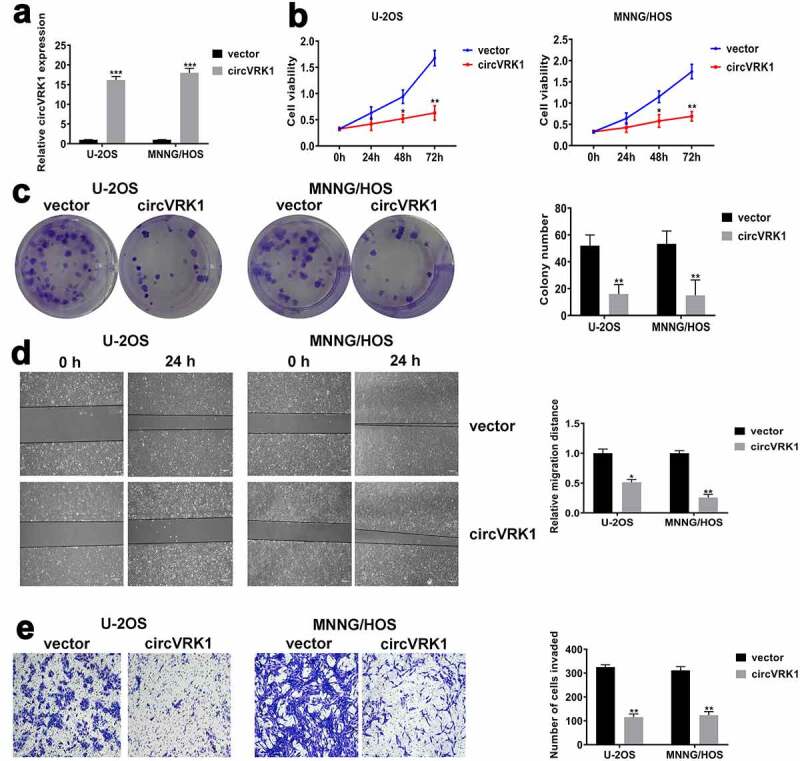
CircVRK1 overexpression inhibited the proliferation, migration, and invasion of osteosarcoma cells

To further investigate the biological functions of circVRK1 in osteosarcoma cells, we overexpressed circVRK1 in U-2OS and MNNG/HOS cells through transfecting circVRK1 overexpression vector and the transfection efficacy was analyzed by qRT-PCR (Figure 3a). Besides, VRK1 mRNA level was not affected by circVRK1 overexpression (Supplementary Figure 1). Subsequently, CCK-8 assay showed that circVRK1 up-regulation significantly decreased the proliferation ability of osteosarcoma cells (Figure 3b). Accordingly, colony formation assay suggested that circVRK1 overexpression obviously repressed the colony-forming ability of osteosarcoma cells (Figure 3c). Moreover, wound-healing and transwell assay exhibited that migration and invasiveness of osteosarcoma cells were remarkably suppressed by the up-regulation of circVRK1 (Figure 3d-e). Together, these experiments disclosed that circVRK1 suppressed the malignant phenotype of osteosarcoma cells.

(A) The overexpression efficiency of circVRK1 was determined by qRT-PCR. (B) CCK-8 assay was employed to detect the cell proliferation activity after circVRK1 overexpression. (C) The colony formation ability of circVRK1 overexpression cells was determined by colony formation assay. (D) The migration ability in circVRK1 overexpressed cells was analyzed by wound-healing assay. Scale bar: 200 μm. (E) Transwell assay was conducted to evaluate the invasiveness after elevating circVRK1 in osteosarcoma cells. Scale bar: 100 μm. *P < 0.05, **P < 0.01, ***P < 0.001.

### CircVRK1 suppresses osteosarcoma growth in vivo

**Figure 4.**
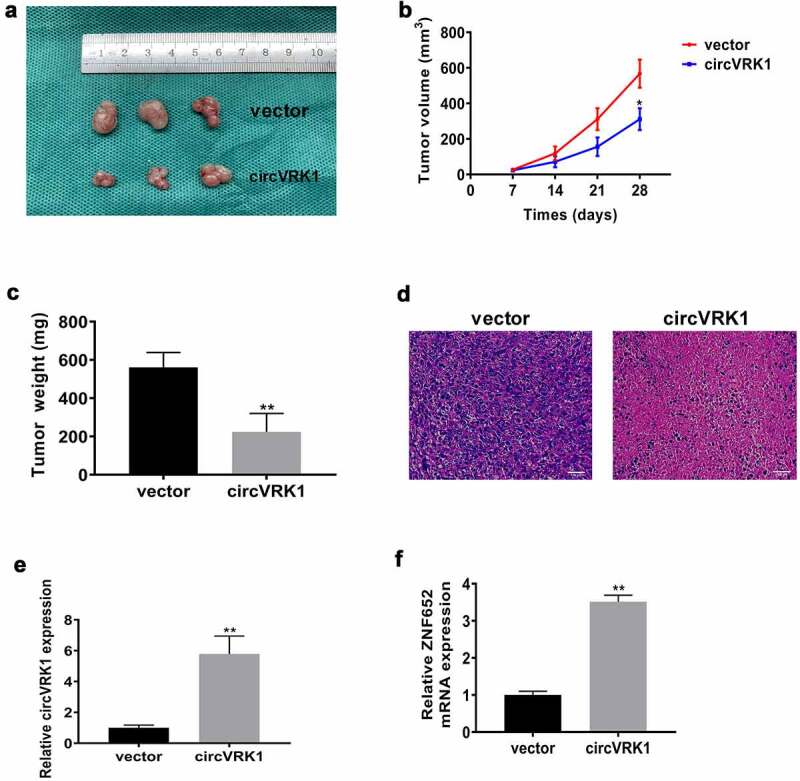
CircVRK1 suppresses osteosarcoma growth in vivo

Then, we investigated the effect of circVRK1 overexpression on osteosarcoma growth in vivo. MNNG/HOS cells transfected with circVRK1 overexpression plasmid or empty vector were subcutaneously injected into the flank of nude mice. Twenty-eight days later, we sacrificed the mice and xenografts were isolated. As illustrated in Figure 4a, circVRK1 overexpression impaired the growth of MNNG/HOS cells in vivo. Additionally, circVRK1 up-regulation results in a dramatic decrease in the final volume and weight of these tumors (Figure 4b-c). The HE staining displayed that there were less malignant cells in the circVRK1 group relative to the control group (Figure 4d). Furthermore, the qRT-PCR results indicated that the levels of circVRK1 and ZNF652 were remarkably elevated in the circVRK1 group relative to those in the control group (Figure 4e-f). Therefore, circVRK1 exhibited tumor suppressing property in vivo.

(A) The image of osteosarcoma xenograft tumors. (B) The growth curve of tumor volume within 28 days. (C) Tumor weight was measured in each group at the ending point. (D) The representative images of hematoxylin–eosin staining of each group. (E-G) The expression levels of circVRK1 as well as ZNF652 mRNA in each group were measured by qRT-PCR. *P < 0.05, **P < 0.01.

### CircVRK1 sponges miR-337-3p in osteosarcoma cells

**Figure 5.**
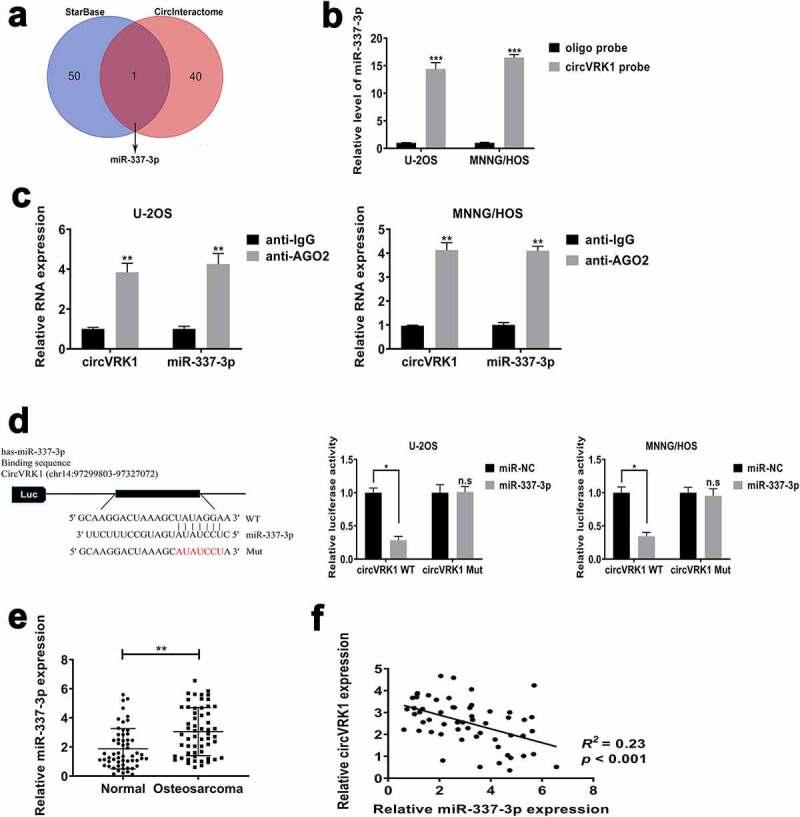
CircVRK1 served as a sponge for miR-337-3p in osteosarcoma cells

Previous researches have demonstrated that many circRNAs play biological functions by adjusting the expression of the target gene through regarding it as a miRNA sponge. Based on previously experimental results that circVRK1 was diffused in the cytoplasm of osteosarcoma cells, we supposed that circVRK1 performed as a miRNA sponge in osteosarcoma cells. We used online database ENCORI Platform (http://starbase.sysu.edu.cn/) and Circular RNA Interactome (https://circinteractome.nia.nih.gov) to search the possible miRNA sponged by circVRK1, and we selected miR-337-3p for in-depth study (Figure 5a). In order to verify the interaction between circVRK1 and miR-337-3p, we initially performed RNA pull-down assay by using the 3' terminal-biotinylated-circVRK1 probe. As illustrated in Supplementary Figure 2, it was verified that the probe could pull-down circVRK1 in osteosarcoma cells and circVRK1 overexpression could increase the efficacy of pull-down. Next, we conducted qRT-PCR analysis and data indicated that miR-337-3p could be pulled-down by the biotinylated-circVRK1 probe in U-2OS and MNNG/HOS cells (Figure 5b). RNA immunoprecipitation assay was further performed to evaluate whether there is a binding relationship between circVRK1 and miR-337-3p. The results showed that circVRK1 and miR-337-3p were dramatically enriched in the AGO2-antibody precipitated RNA fraction in osteosarcoma cells (Figure 5c). The results of dual-luciferase reporter assay furthermore exhibited that the activity of luciferase of wild-type vector containing circVRK1 sequences was declined by the up-regulation of miR-337-3p, but the luciferase activity of mutant vector in U-2OS and MNNG/HOS cells was not affected by miR-337-3p mimics (Figure 5d). Additionally, we evaluated miR-337-3p levels of clinical samples, the results presented that miR-337-3p abundance in osteosarcoma tissues was remarkably increased, contrary to normal tissues (Figure 5e), and it was noted that the level of miR-337-3p was adversely related to the expression of circVRK1 in osteosarcoma tissues (figure 5f). Based on the above, circVRK1 served as a sponge for miR-337-3p in osteosarcoma cells.

(A) Venn diagram showed the overlapping of two databases (StarBase and CircInteractome). (B) The level of miR-337-3p was quantified by qRT-PCR after biotinylated-circVRK1 pull-down in osteosarcoma cells. (C) RNA-immunoprecipitation in U-2OS and MNNG/HOS cells using anti-AGO2 antibody or anti-IgG antibody. CircVRK1 and miR-337-3p abundance in the immune-precipitated fraction was measured by qRT-PCR. Results are presented relative to IgG immunoprecipitation, set as 1. (D) The left panel exhibited the binding site between circVRK1 and miR-337-3p. Luciferase activity was measured in U-2OS and MNNG/HOS cells co-transfected with WT circVRK1 or Mut circVRK1 and miR-NC or miR-337-3p, the results were showed in the right panel. (E) The levels of ZNF652 in osteosarcoma tissues and matched normal tissues were measured by qRT-PCR. (F) The association between the expression of miR-337-3p and circVRK1 in osteosarcoma tissues was assessed by Pearson’s correlation coefficient. *P < 0.05, **P < 0.01, ***P < 0.001, n.s no significance.

### CircVRK1 promotes ZNF652 expression by sponging miR-337-3p in osteosarcoma cells

**Figure 6.**
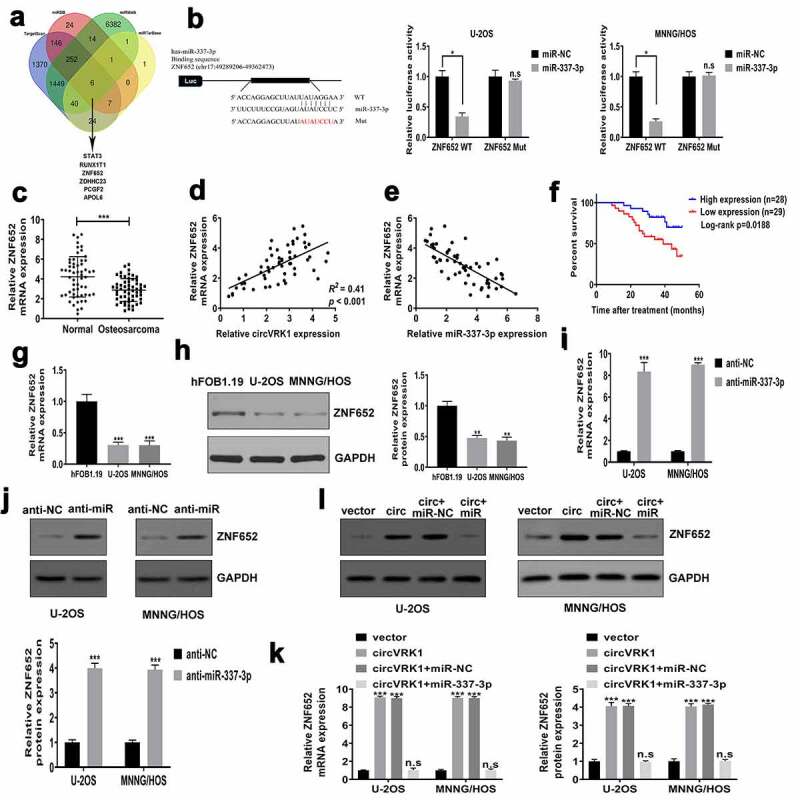
CircVRK1 promotes ZNF652 expression by regulating miR-337-3p in osteosarcoma cells

To dissect the ceRNA network regulated by circVRK1, the downstream target of miR-337-3p was forecasted by online databases TargetScan (http://www.targetscan.org/), miRDB (http://mirdb.org/), miRWalk (http://mirwalk.umm.uni-heidelberg.de/), and miRTarBase (http://mirtarbase.mbc.nctu.edu.tw/). As shown in Figure 6a, we screened six overlapping genes: STAT3, RUNX1T1, ZNF652, ZDHHC23, PCGF2 and APOL6. We conducted qRT-PCR analysis and found that only ZNF652 increased was consistent with the overexpression of circVRK1 in U-2OS and MNNG/HOS cells (Supplementary Figure 3), so we selected ZNF652 for further experiments. The dual-luciferase reporter assay indicated that luciferase activity of vector including the wild-type 3'-UTR of ZNF652 mRNA was declined by miR-337-3p mimics. However, miR-337-3p overexpression did not influence the luciferase activity of mutant vector in osteosarcoma cells (Figure 6b). The results of qRT-PCR demonstrated that ZNF652 expression in osteosarcoma tissues were dramatically reduced in osteosarcoma tissues contrast to that in noncancerous tissues (Figure 6c). Significant positive correlation was found between circVRK1 and ZNF652 in osteosarcoma samples (Figure 6d), while miR-337-3p was negatively correlated with ZNF652 (Figure 6e). The survival analysis revealed that patients with low ZNF652 levels had poorer clinical outcome than patients in the high ZNF652 expression group (figure 6f). Meanwhile, qRT-PCR and western blot assay displayed that the expression of ZNF652 in U-2OS and MNNG/HOS cells were evidently decreased than that in osteoblast cells (Figure 6g-h). Moreover, the effect of miR-337-3p inhibitor on ZNF652 expression was investigated. MiR-337-3p knockdown greatly elevated abundances of ZNF652 in U-2OS and MNNG/HOS cells (Figure 6i-j). Besides, ZNF652 expression was dramatically increased by circVRK1 overexpression, whereas this promotion effect was attenuated by miR-337-3p up-regulation in U-2OS and MNNG/HOS cells (Figure 6k-l). Together, circVRK1 facilitated the expression of ZNF652 by regulating miR-337-3p in osteosarcoma cells.

(A) Venn diagram showed the overlapping genes of four databases (TargetScan, miRDB, miRWalk and miRTarBase), and STAT3, RUNX1T1, ZNF652, ZDHHC23, PCGF2 and APOL6 were discovered to be the candidate target gene of miR-337-3p. (B) The left panel showed the binding site between miR-337-3p and ZNF652. The luciferase activity was measured in U-2OS and MNNG/HOS cells co-transfected with ZNF652 3'-UTR-WT or ZNF652 3'-UTR-Mut and miR-NC or miR-337-3p, and the results were showed in the right panel. (C) The levels of ZNF652 in osteosarcoma tissues and matched normal tissues were determined by qRT-PCR. (D-E) The association between the level of ZNF652 mRNA and circVRK1 or miR-337-3p in osteosarcoma tissues was assessed by Pearson’s correlation coefficient. (F) The relationship between ZNF652 expression and osteosarcoma patients’ overall survival was analyzed by Kaplan-Meier analysis. (G-H) The mRNA and protein levels of ZNF652 in osteosarcoma cells and osteoblasts were measured by qRT-PCR and western blot. (I-J) The mRNA and protein levels of ZNF652 were measured in U-2OS and MNNG/HOS cells transfected with anti-miR-337-3p or anti-NC by qRT-PCR and western blot. (K-L) The impacts between circVRK1 overexpression and miR-337-3p mimics on ZNF652 expression were unveiled by qRT-PCR and western blot. *P < 0.05, **P < 0.01, ***P < 0.001, n.s is no significance.

### CircVRK1 inhibits osteosarcoma progression by regulating miR-337-3p and ZNF652

**Figure 7.**
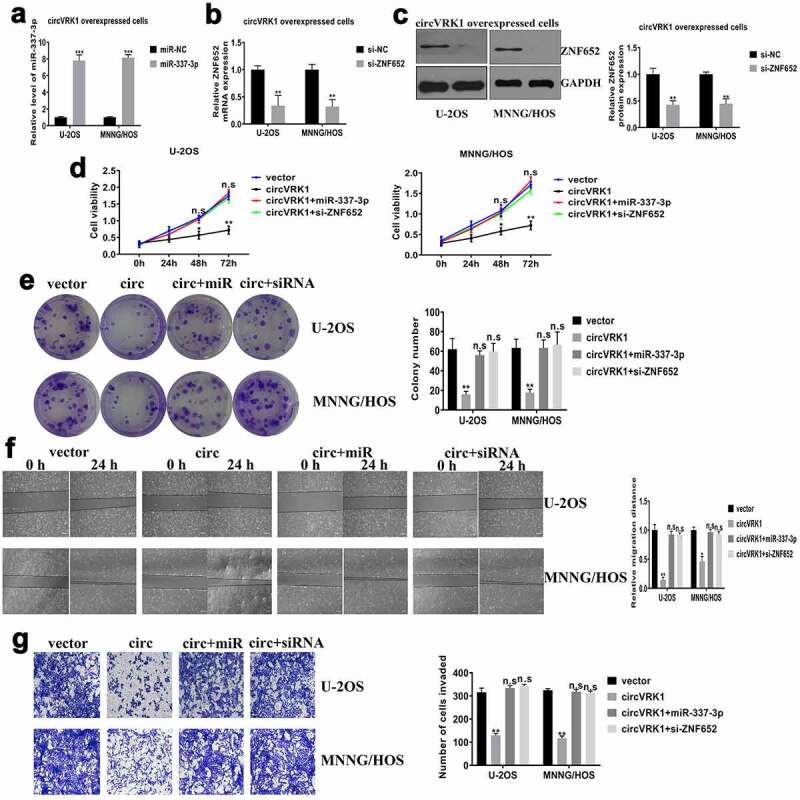
CircVRK1 overexpression inhibits osteosarcoma progression by regulating miR-337-3p and ZNF652

In order to verify whether circVRK1 affected osteosarcoma progression was mediated by the miR-337-3p/ZNF652 axis, we transfected miR-337-3p mimics or si-ZNF652 in circVRK1 overexpressed cells, respectively (Figure 7a-c). Subsequently, we performed cell proliferation and colony formation assays to assess cell viability. The results presented that miR-337-3p mimics or ZNF652 knockdown abrogated the suppressive effect of circVRK1 on proliferation and colony-forming of osteosarcoma cells (Figure 7d-e). Meanwhile, wound-healing and transwell assays manifested miR-337-3p overexpression or ZNF652 depletion abolished the impaired capacities of migration and invasion caused by circVRK1 in osteosarcoma cells (figure 7f-g). Overall, these findings demonstrated that circVRK1 inhibited the progression of osteosarcoma by modulating the miR-337-3p/ZNF652 axis.

(A) The transfection efficiency of miR-337-3p mimics in circVRK1 overexpressed osteosarcoma cells was measured by qRT-PCR. (B-C) The transfection efficiency of si-ZNF652 in overexpressed osteosarcoma cells was determined by qRT-PCR and western blot. (D) CCK-8 assay was performed to investigate the impacts between circVRK1 overexpression and miR-337-3p mimics or ZNF652 depletion on cell proliferation in osteosarcoma cells. (E) The effects between circVRK1 and miR-337-3p mimics or ZNF652 silencing on cell colony-forming ability were revealed by colony formation assay in U-2OS and MNNG/HOS cells. (F-G) Wound-healing assay and transwell assay were employed to display the influences between circVRK1 and miR-337-3p mimics or ZNF652 absence on the migration and invasion of U-2OS and MNNG/HOS cells. *P < 0.05, **P < 0.01, n.s is no significance.

## Discussion

CircRNAs have been considered as a by-product of molecular splicing since they were observed in eukaryotic cells 40 years ago [15,41]. However, in recent years, a growing body of circRNAs have been found owing to the application of high-throughput sequencing, and our comprehension of circRNAs has gradually improved. A tremendous amount of circRNAs have been detected in tissues and cells of different human organs, most of which are more stable and abundant than their linear isoform [42]. Subsequent researches have confirmed that circRNAs are dysregulated in various diseases and could be served as a promising diagnostic and prognostic biomarker for cancers [43,44]. For example, in osteosarcoma, increased hsa_circ_001621 could predict the poor prognosis, and patients with higher circMYO10 expression had a worse clinical outcome [45,46]. CircVRK1 is a novel discovered circular RNA, which has been pinpointed as a key anti-oncogene in the breast cancer and esophageal cancer [19,20]. The current study initially showed that circVRK1 expression in osteosarcoma tissues and cell lines was more down-regulated than that in normal tissues and osteoblasts, and the low circVRK1 was correlated with the distant metastasis, which provided a basis for the poor prognosis in osteosarcoma patients. Furthermore, nucleoplasmic separation assay, RNase R treatment analysis and RNA stability assay indicated that circVRK1 was located in the cytoplasm of osteosarcoma cells and possessed a stable circular structure. In addition, in vitro assays revealed that circVRK1 overexpression significantly inhibited proliferation, migration, and invasion of osteosarcoma cells, and in vivo experiment indicated that circVRK1 repressed osteosarcoma growth. Our findings suggested that circVRK1 served as a suppressive role in osteosarcoma.

Recently, accumulating studies demonstrated that circRNAs exert their biological roles as competitive endogenous RNAs during the tumorigenesis [47,48]. For example, circZNF609 depletion repressed nasopharyngeal carcinoma cells proliferation via sponging miR-188 [49]. Hsa_circ_0060745 boosts the progression of colorectal cancer through miR-4736 sponging [50]. In osteosarcoma, hsa_circ_0001105 impairs cell proliferation and metastasis by sponging miR-766 [51], and hsa_circ_0000285 acts as a ceRNA for miRNA-599 to facilitate osteosarcoma progression [52]. Similar to the above studies, we have confirmed the ‘miRNA sponge’ role of circVRK1 in osteosarcoma cells. The online databases StarBase and CircInteractome were used for bioinformatics analysis to find microRNAs that may interact with circVRK1, and finally miR-337-3p was selected as our research target. In previous studies, miR-337-3p was considered to play a tumor suppressive effect in some malignant tumors [53–55], but its role in osteosarcoma has not been revealed. At present, this study indicated that miR-337-3p was increased in osteosarcoma tissues and cells, further luciferase reporter, RNA pull-down, and RIP assays verified the relationship between circVRK1 and miR-337-3p. Rescue experiments illustrated that miR-337-3p overexpression attenuated the effects of circVRK1 on cell proliferation, colony-forming, migration, and invasiveness of osteosarcoma cells.

In order to investigate the ceRNA network mediated by circVRK1 thoroughly, this study forecasted the possible target genes of miR-337-3p depend on databases and found that 3'-UTR of ZNF652 mRNA had miR-337-3p binding sites. ZNF652 is a novel zinc-finger protein that has been identified as a transcriptional repressor and directly repressed invasion and metastasis in breast cancer [31]. Currently, we found that ZNF652 abundance was obviously decreased in osteosarcoma samples and cells, and ZNF652 expression was adversely related to miR-337-3p, whereas positively related to circVRK1 level in osteosarcoma tissues. Additionally, the expression of ZNF652 was adjusted by circVRK1 and miR-337-3p in osteosarcoma cells, and the interaction between ZNF652 and miR-337-3p was verified by the luciferase reporter assay. Furthermore, we transfected si-ZNF652 into circVRK1 overexpression cells and uncovered that ZNF652 knockdown abolished the impact of circVRK1 on the malignant phenotype of osteosarcoma cells.

## Conclusion

In summary, our work demonstrated that circVRK1 expression was frequently reduced in osteosarcoma and low level of circVRK1 was related to tumor metastasis and poor prognosis. From the perspective of mechanism, circVRK1 served as a molecule sponge for miR-337-3p and mediated the ceRNA network to promote the expression of ZNF652, finally suppressed osteosarcoma progression. Our findings might provide a novel and promising strategy for the treatment of osteosarcoma.

## Limitation of the study

Any research has its own limitations and shortcomings, and this study is no exception. Osteosarcoma is the most common malignant bone tumor, but the number of cases collected by our department is not large. Besides, owing to the ineffective preservation of tissue specimens and some cases lost, only 57 cases were selected for this study. Therefore, there are not enough cases to analyze and verify the relationship between circVRK1 and osteosarcoma progression. However, this work still provides valuable guidance for our future research. In the follow-up study, we will join the multi-center research to obtain a large amount of osteosarcoma cases to further investigate the clinical significance of circVRK1 and develop precise therapeutic drugs for osteosarcoma targeting circVRK1.

## Supplementary Material

Supplemental Material

## Data Availability

The datasets used in the current study are available from the corresponding authors on reasonable request.
