# Supplementary material for: Circular RNA circVRK1 suppresses the proliferation, migration and invasion of osteosarcoma cells by regulating zinc finger protein ZNF652 expression via microRNA miR-337-3p
Source: Bioengineered. 2021 Aug 23;12(1):5411–27. doi: 10.1080/21655979.2021.1965695 (PMC8806728; doi:10.1080/21655979.2021.1965695)
Supplement: Supplemental Material [file KBIE_A_1965695_SM6348.zip › Supplementary file.docx]

**Supplementary Figure 1**


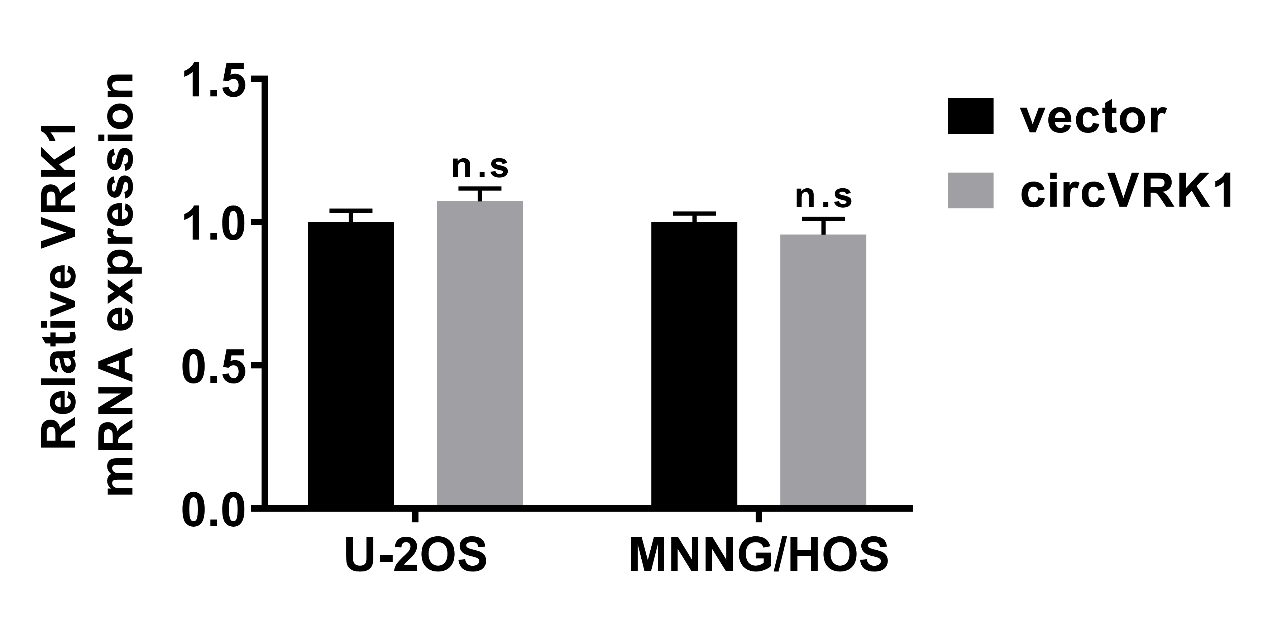


**Supplementary Figure 2**


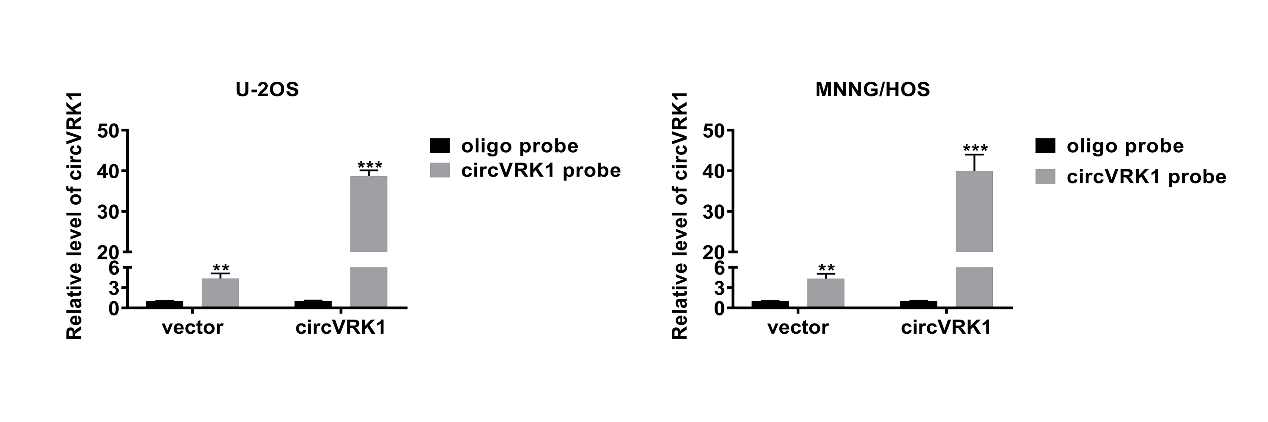


**Supplementary Figure 3**


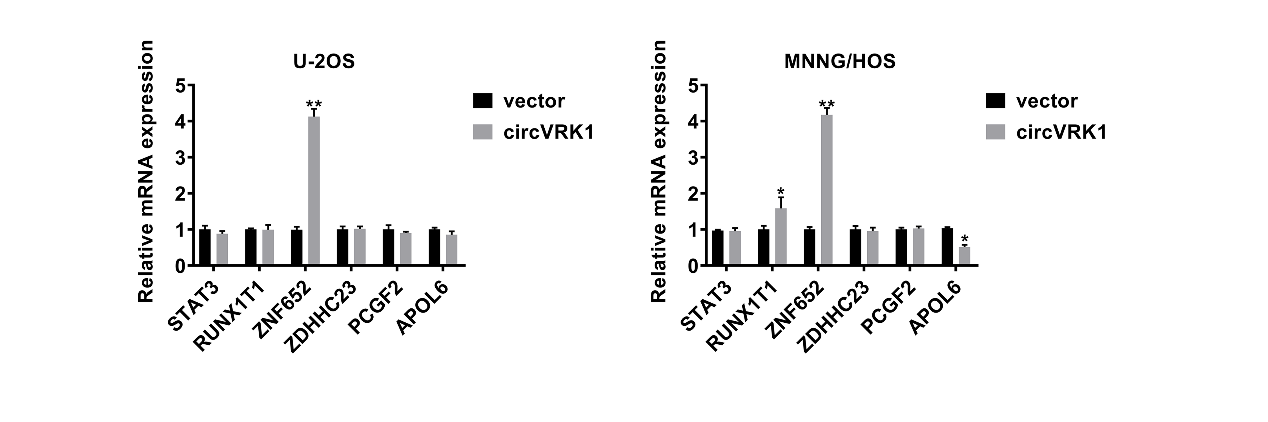


**Supplementary Figure Legends**

**Supplementary Figure 1**

qRT-PCR was performed to detect the effect of circVRK1 overexpression on VRK1 mRNA level. n.s no significance.

**Supplementary Figure 2**

Lysates from U-2OS and MNNG/HOS cells transfected with circVRK1 overexpression vector or empty vector were subjected to biotinylated-circVRK1 pull-down assay and the levels of circVRK1 were measured by qRT-PCR. ***P*<0.01, ****P*<0.001.

**Supplementary Figure 3**

The relative mRNA expression of STAT3, RUNX1T1, ZNF652, ZDHHC23, PCGF2, APOL6 was measured by qRT-PCR in osteosarcoma cells transfected with circVRK1 overexpression vector. **P*<0.05, ***P*<0.01.
